# The Perception of a Three-Dimensional-Printed Heart Model from the Perspective of Different Stakeholders: A Complex Case of Truncus Arteriosus

**DOI:** 10.3389/fped.2017.00209

**Published:** 2017-09-28

**Authors:** Giovanni Biglino, Sarah Moharem-Elgamal, Matthew Lee, Robert Tulloh, Massimo Caputo

**Affiliations:** ^1^School of Clinical Sciences, Bristol Heart Institute, University of Bristol, Bristol, United Kingdom; ^2^University Hospitals Bristol, NHS Foundation Trust, Bristol, United Kingdom; ^3^National Heart Institute, Giza, Egypt

**Keywords:** truncus arteriosus, congenital heart disease, three-dimensional printing, three-dimensional reconstruction, case report

## Abstract

The case of an 11-year-old male patient with truncus arteriosus is presented. The patient has a right aortic arch, a repaired truncus arteriosus, pulmonary artery stenosis, as well as conduit stenosis, with a complex surgical plan being discussed. In order to gather additional insight into the patient’s anatomy prior to the surgery and to facilitate communication with the patient’s parents, a three-dimensional (3D) model of his heart and main vessels was created from computed tomography data. Feedback was collected from different stakeholders. The patient and his parents were both struck by the size of the heart, with the parents further elaborating on how the 3D model was more intuitive a tool than medical images as well as “*an helpful talking point to the other members of the family*” and potentially also at school. The surgeon and cardiologist commented on gaining better understanding of the 3D relationship between a markedly narrowed right pulmonary artery and the aorta, with the surgeon ultimately coming to a decision of dividing the ascending aorta quite high to access the right pulmonary artery for patch reconstruction and thus planning to arrest the circulation beforehand. The imaging expert remarked on the potential to “*improve communication in multidisciplinary meetings*,” while a medical trainee, who also had a chance to evaluate the model, remarked that “*having the model in front of me and being able to see the exact abnormality makes this particular case much more memorable. [*…*] 3D printed models could have immense potential in pathology and anatomy teaching for the training of healthcare professionals*.”

## Introduction

Three-dimensional (3D) printing technology is gaining increased interest in the field of congenital heart disease (CHD). Advocated beneficial applications include aiding in the surgical decision-making process, testing novel devices such as stents, and facilitating the communicative process between patients (and families) and cardiologists during clinical consultations (1–5). Two important elements can eventually lead to translate this rapidly evolving technology into clinical practice: (a) gathering evidence of the effectiveness of the models on a large scale and (b) considering the perspective of all stakeholders in the technology, according to a model of social construction of technology (6). Stakeholders in the context of using 3D models of CHD in clinical practice include the surgeon, the cardiologist, the imaging expert who provides the data for creating the model and may be involved in discussing the case, trainees who may learn from practicing on a complex CHD case, and crucially the patients and their families. Here, we present a case study where a 3D heart model was manufactured to assess a complex case of truncus arteriosus and feedback on the 3D model itself was gathered from multiple users.

## Case Report

The patient (11-year-old, male) has a diagnosis of postoperative truncus arteriosus, with residual truncal regurgitation, and cardiac conduit stenosis. The patient has a right aortic arch, a repaired ventricular septal defect (VSD) and branch pulmonary artery stenosis. The surgical plan being discussed included: pulmonary arterioplasty, patch enlargement of the trunk as well as the right and left pulmonary arteries, relief of right ventricular outflow tract obstruction, replacement of the right ventricle to pulmonary artery conduit, truncal valve cusp repair, and interposition graft in the ascending aorta. It was deemed that a 3D model would be helpful to appreciate the size and position of the pulmonary arteries with respect to the conduit, as well as to illustrate the surgical plan to the patient and his family.

## 3D Model

**Figure 1:**
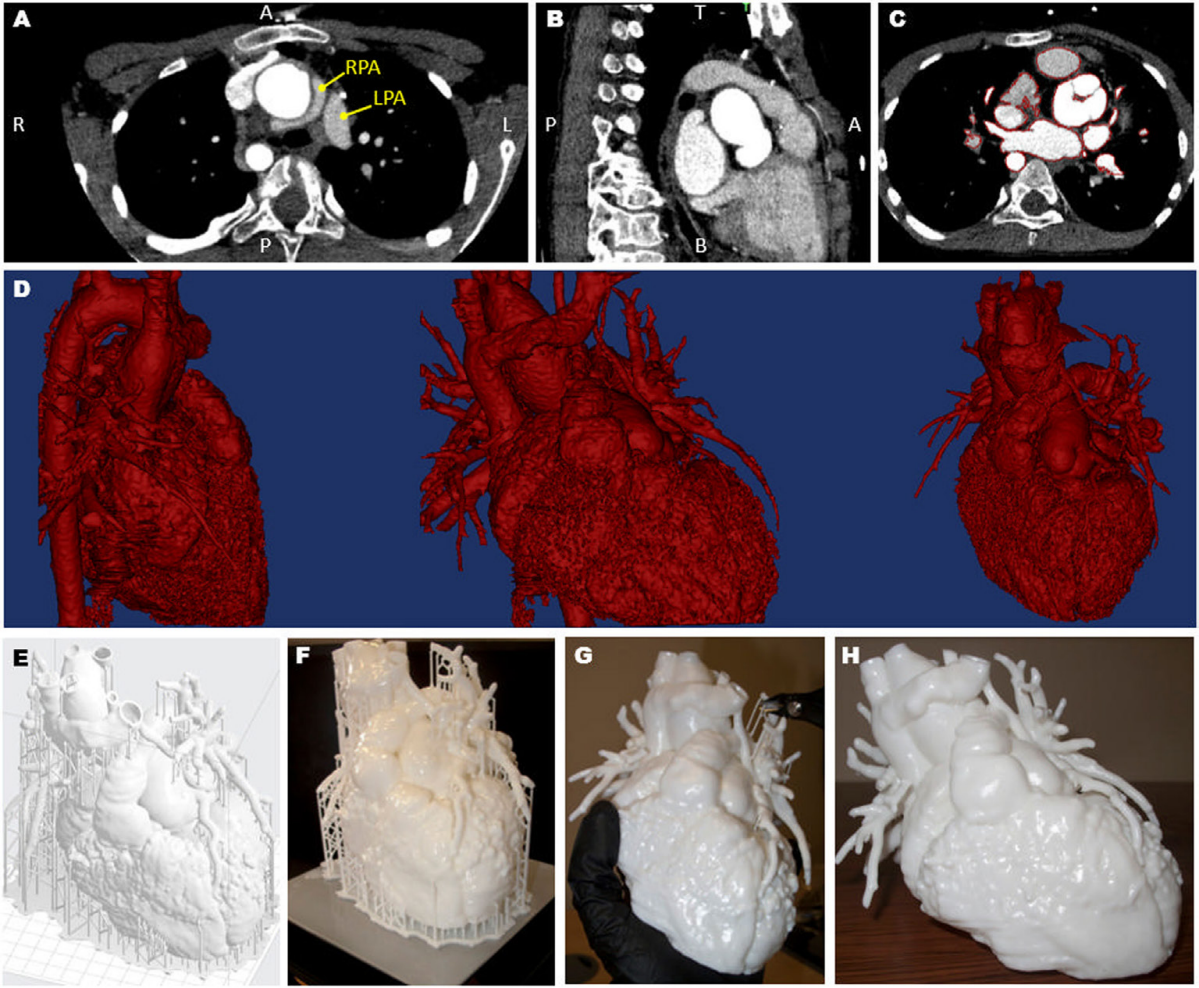
Views of the computed tomography imaging dataset **(A–C)** including highlight of the position of the left and right pulmonary artery (LPA and RPA), showing the RPA wrapping around the aorta **(A)**; the red outline in panel **(C)** shows the regions included in the three-dimensional (3D) model; the data are then rendered in 3D [**(D)**, showing different views]; a scaffold **(E)** is built around the model prior to 3D printing **(F)** and the removed **(G)**, resulting in the final patient-specific model **(H)**.

A 3D model was created from computed tomography data acquired 6 months prior to the surgery (Siemens Medical Solutions, Erlangen, Germany). Images were processed using commercial software (Mimics Research 19.0, Materialise NV, Leuven, Belgium) (7) and the resulting 3D surface was further processed, smoothed, and extruded with a 1.2-mm wall thickness (3-matic Research 11.0, Materialise). The obtained surface file was further processed using the 3D printer software (PreForm 2.10.3, Formlabs Inc., Somerville, MA, USA), which allowed a scaffold to be automatically created around the model for facilitating the printing process. The model was printed in white resin (Form2, Formlabs Inc.). Printing time was 27 h, after which time the scaffold had to be removed. The whole process (Figure 1) was undertaken at our center, with the 3D printer being available in house.

## Feedback from Stakeholders

Here, we present feedback that was collected from different stakeholders either verbally or *via* email. Permission was received to record conversations and quote them in an anonymous form. A draft of the manuscript was shared with the family prior to submission and written informed consent was obtained for the publication of this case report.

### The Parents’ Perspective

The father of the patient commented very positively on the 3D model. “*[The surgeon] present[ed] us with the 3D model [*…*] and used that to help and explain exactly what he needed to do and why he needed to do it, so it [was] really fascinating to see it, really helpful. The first thing I noticed was how big it was, and I hadn’t comprehended how big the heart was, [*…*] how it actually fitted in [my son]. To be able to turn it round and notice how the pulmonary arteries were narrowed, [*…*] to actually see it in front of you it was fantastic*.” He also commented on the usefulness of the model compared to medical imaging: “*We had an echo [*…*] and in that image, I think it’s a 3D image, you can see the valve working, and that was incredible, because I’ve never seen one that clear. [*…*] But even that it is difficult to comprehend*… *The doctors are talking about, you know, left pulmonary artery, and you can’t really imagine it in your head*… *So the model had been brilliant*.” He remarked on the usefulness of potentially having follow-up models to highlight differences before/after surgeries/procedures. “*What I would find amazing is to have a 3D model of [my son’s] heart [as a baby], and then ideally one now, so we can show him exactly why he has to go through the surgery, and what’s happened as a result of the surgery*.” He also mentioned the usefulness and the potential of the model for communicating with others: “*[*…*] it’s just an helpful talking point to the other members of the family*” *and* “*I think it would be brilliant to take it to the school and explain and have a lesson about his heart*.” Labeling was indicated as a potential improvement: “*One thing that I think it would be helpful in the future, I don’t know whether that is possible, is to label the different tubes. [The surgeon] told us in the consultation what they were, and [my wife] has got a great idea of how it all works, so we can kind of piece it together afterwards, but I guess some labeling would be good*.”

### The Patient’s Perspective

The conversation with the patient was shorter but when asked if he found the model interesting he answered affirmatively, and equally when asked if he felt that the model helped him to understand why he needed the operation he responded affirmatively. The patient also appeared to be particularly struck by the size of his own heart and said: “*When I saw it, when I saw the size of it, [and] when you see it next to me*… *it doesn’t really fit*.”

### The Surgeon’s Perspective

The cardiac surgeon operating on the case emphasized that “*this was a very complex re-operation and all the pre-operative investigations did help significantly to plan the surgical strategy*.” In terms of the 3D heart model, he commented that its “*added value [*…*] was a better understanding of the three-dimensional relationships of the very narrowed right pulmonary artery and the dilated ascending aorta*.” In fact “*during the operation a decision had to be made regarding the access to the distal RPA. Having had the 3D model assessed pre-operatively it was clear [*…*] that the ascending aorta had to be divided quite high in order to access the RPA for the patch reconstruction. This meant that we could plan the arrest of the circulation beforehand (which otherwise it is not usually needed in this type of re-operation and not easily anticipated from the routine pre-operative investigations)*.” He also noted that the model could “*help facilitating the communication in the operating room*.”

### The Cardiologist’s Perspective

The model was also helpful for the cardiologist “*to further understand the anatomical relationships of the different structures, the branch pulmonary arteries and the aortic trunk*” and “*to be able to demonstrate to the surgeon images from echocardiography which would be helpful in pre-and post-operative planning*.”

### The Imager’s Perspective

The imager commented on the fact that models “*provide with a different perspective of the patient’s heart. Instead of viewing the heart and making measurements from different views on a monitor, 3D heart models provide an added perception to size and anatomical relationships*” and felt that they have “*the potential to assist discussions and improve communication in multidisciplinary meetings between pediatric cardiologist, cardiac surgeon and the cardiac imager to better decision-making and ultimately offer the patient the best possible management plan*.” The imager agreed that models can be helpful when communicating with families, to better show “*what to expect from the management plan*.”

### The Trainee’s Perspective

A third year medical student also assessed the model and remarked that “*as someone who has medical knowledge but does not have much experience of viewing scans, the 3D models clarifies the spatial and anatomical variations of the heart*” allowing “*to see the exact points where the aorta and pulmonary artery are connected*.” As a student, he found the model “*more meaningful than a 2D picture [*…*] to spot the abnormal anatomy*” and interestingly noted: “*having the model in front of me and being able to see the exact abnormality makes this particular case much more memorable. When I am confronted with a case of truncus arteriosus in the future, I will be able to recall this heart model and think of the exact abnormality in my head. This is important as in medical school we are presented with so many different diseases that it is hard to remember what everything is*.” As such, “*3D printed models could have immense potential in pathology and anatomy teaching for the training of healthcare professionals*.”

## Discussion and Conclusion

The effectiveness of using 3D cardiac models for the purpose of planning or practicing procedures still necessitates evidence on a large scale, but small studies are beginning to indicate scenarios where patient-specific 3D models can play a useful role in clinical practice. A recent study, for instance, focused on complex patients with uncertain optimal management strategy, presenting six models derived from patients with complex muscular VSDs and double-outlet right ventricle, and concluded that using the models can improve spatial appreciation of complex anatomical structures and can play a role for pre- and intra-interventional management (8). Another interesting study aimed to assess the impact on surgical decision-making using 3D models in a small heterogeneous patient cohort (two complex double-outlet right ventricles, two criss-cross atrioventricular connections, one congenitally corrected transposition of great arteries with pulmonary atresia), and concluded that all surgeries were performed successfully with 3D models augmenting the existing available clinical data (9). Case studies have also presented successful applications of 3D models in the clinical management of congenital cases, including compelling examples such as:

- evaluating the case of a 6-month-old patient with pulmonary atresia, VSD, complete agenesis of the pulmonary trunk and absence of right and left pulmonary arteries (10);
- planning stent angioplasty of a pulmonary venous baffle obstruction in a 30-year-old man with transposition of the great arteries (Mustard repair) (11);
- simulating endovascular stenting of the hypoplastic arch in a 15-year-old patient with repaired aortic coarctation (12).

In this context, the present report illustrates how an in-house manufactured 3D model was helpful both for the surgeon and cardiologist to gain additional insight into the anatomy prior to the procedure, as well as for the parents and the patient to appreciate key concepts such as the size of the heart, the caliber of the vessels involved in the procedure and their position relative to each other. The feedback collected for this case also highlighted the value of the patient-specific model for improving training and communication among professionals, in agreement with recent observations (13–16).

Each case possesses unique elements, in terms of the dynamics at play, the clinical history of the patient and the perspective of those involved. Individual accounts should be “respected and recognized and hailed as significant” (17). So, while large and, where possible, randomized studies are essential to gather statistical evidence, we should remain aware of the complementary possibility of learning from the evidence of the singular (17). Individual experiences can provide with precious insight into mechanisms underlying the use of this exciting technology in clinical practice, including any potential anxieties, unexpected responses (particularly from young people), and areas of improvement from the perspective of medical staff and patients. Further research in this context is certainly warranted.

## Author Contributions

All authors contributed to drafting and revising the manuscript. The heart model was created by GB.

## Conflict of Interest Statement

The authors declare that the research was conducted in the absence of any commercial or financial relationships that could be construed as a potential conflict of interest.
